# Acute Coronary Syndromes in the Elderly

**DOI:** 10.12688/f1000research.11064.1

**Published:** 2017-10-02

**Authors:** Niels Engberding, Nanette K. Wenger

**Affiliations:** 1 Department of Medicine, Division of Cardiology, National Jewish Health, Denver, Colorado, USA; 2 Emory Heart and Vascular Center, Atlanta, Georgia, USA; 3 Department of Medicine, Division of Cardiology, Emory University School of Medicine, Atlanta, Georgia, USA

**Keywords:** acute coronary, elderly, cardiovascular disease

## Abstract

The clinical evidence for treatment of acute coronary syndrome (ACS) in the elderly is less robust than in patients younger than 75 years. The elderly have the highest incidence of cardiovascular disease and frequently present with ACS. This number can be expected to increase over time because society is aging. Older adults often sustain unfavorable outcomes from ACS because of atypical presentation and delay in recognition. In addition, elderly patients commonly do not receive optimal guideline-directed ACS treatment. Owing to their high baseline risk of ischemic complications, the elderly also fare worse even with optimal ACS treatment as they frequently have more complex coronary disease, more comorbidities, less cardiovascular reserve, and a higher risk of treatment complications. They are also subjected to a broader range of pharmacologic treatment. Treatment complications can be mitigated to some extent by meticulous dose adjustment of antithrombotic and adjunctive therapies. While careful transitions of care and appropriate utilization of post-discharge secondary preventive measures are important in ACS patients of all ages, the elderly are more vulnerable to system errors and thus deserve special attention from the clinician.

## Epidemiologic data

Elderly patients (>75 years of age) ^1^ constitute a large proportion of those patients presenting with acute coronary syndrome (ACS), and temporal trends in the incidence of myocardial infarction document a shift toward older adults ^2^. The average ages at first ACS presentation in the US are 65 years for men and 72 years for women. About two thirds of myocardial infarctions occur in patients older than 65 years of age, and one third in patients older than 75 years of age. Randomized clinical trials, on the other hand, have included substantially fewer elderly patients than clinicians encounter in real life ^3^. Thus, the basis of evidence forming the foundation of ACS treatment may not apply to a large number of patients, and clinicians need to extrapolate evidence to match their older patients’ needs and preferences. Sixty percent of ACS hospitalizations occur in patients older than 65 years, and 85% of ACS mortality occurs in the Medicare population. Most deaths related to myocardial infarction occur in patients older than 65 years of age ^4^.

Age is not only a powerful risk factor for cardiovascular disease; it is also an independent risk factor for adverse outcomes after cardiovascular events, for complications of cardiovascular procedures and interventions, and for side effects of pharmacotherapy, particularly from antithrombotic therapies. The mortality rate after a first non-ST segment elevation myocardial infarction (non-STEMI) in very elderly patients is very high: with respect to 1-year outcomes, among patients who were 65–79, 80–84, 85–89, and at least 90 years old, mortality increased progressively from 13.3% to 23.6%, 33.6%, and 45.5%, respectively ^5^.

In addition, older patients generally have more complex cardiovascular disease, more comorbidities, and generally a more atypical clinical presentation. There is a greater prevalence of hypertension, congestive heart failure (CHF), atrial fibrillation, cerebrovascular disease, anemia, and renal insufficiency in older patients with ACS. Age also has important implications on pharmacokinetics and pharmacodynamics ^6^. Challenges in taking care of elderly patients with ACS include timely recognition, not withholding lifesaving therapies on the basis of age alone, and respecting the patients’ preferences and goals of care.

## Atypical symptoms

There may be several explanations for why the elderly have worse outcomes with ACS. While chest pain remains the most common presentation for ACS, elderly patients frequently present with atypical symptoms (meaning, without chest pain) ^7^. In patients who present without chest pain, the diagnosis of ACS is often missed or delayed, leading to worse outcomes. Notably, chest pain as a presenting symptom occurs in only 40% of patients older than 85 years but is present in nearly 80% of patients under 65 years. Common symptoms in the elderly presenting with ACS include dyspnea, diaphoresis, nausea and vomiting, and syncope. In patients at least 85 years old, an atypical presentation of myocardial infarction appears to be the standard and the clinician must be prepared to diagnose ACS in many acutely ill patients of this age ^8^. Acute pulmonary edema is more commonly a presentation of the elderly patient with ACS. Increased arterial stiffness as manifested with increased arterial pulse pressure as well as increased prevalence of multivessel coronary artery disease (CAD) may explain why older patients with ACS are more likely to present with signs and symptoms of CHF ^9^.

Aside from atypical symptoms, the 12-lead electrocardiogram (ECG), a standard investigation in patients with suspected ACS, may be non-diagnostic and therefore serial ECGs are recommended to diagnose high-risk findings such as ST segment elevation. The diagnosis of a STEMI is more challenging in patients presenting with left bundle branch block (LBBB). Therefore, the higher prevalence of LBBB in the elderly may contribute to diagnostic uncertainty in the early phase of presentation, when rapid risk stratification and triage are most important.

Prehospital delays also contribute to prevent prompt treatment. Despite having more severe coronary disease than younger patients at coronary angiography, elderly patients are more likely to be treated medically and experience more adverse outcomes ^10^. Additionally, the hemodynamic impact of a given infarct size may be more pronounced in the elderly because of reduced cardiac reserve. The age-related decline in cardiac reserve may be related to reduced beta-adrenergic responsiveness. There is also a higher likelihood of comorbid illnesses with advancing age. Not only do these comorbidities obscure the presentation of ACS, they also contribute to worse outcomes. Type 2 myocardial infarctions, which result from increased myocardial oxygen demand in the setting of severe fixed obstructive CAD, are commonly caused by comorbidities such as tachycardia, pneumonia with hypoxemia, chronic pulmonary disease, and bleeding episodes. These comorbidities frequently complicate hospital admissions of elderly patients and need to be recognized since they require treatment strategies different than those of type 1 myocardial infarctions. In general, elderly patients are more likely to experience complications of ACS, such as CHF, heart block, ventricular rupture, and atrial fibrillation. Furthermore, frailty and disability can complicate acute hospitalization as well as convalescence and rehabilitation. On the other hand, frailty is not considered in clinically accepted risk scores.

## General considerations in management

The treatment of ACS has evolved significantly over the past 40 years. Classically, ACS is caused by thrombotic obstruction of an epicardial coronary artery. Thus, treatment focuses primarily on early coronary revascularization supported by the use of antithrombotic pharmacotherapy ^11^. In general, more aggressive use of invasive coronary procedures and antithrombotic medications is associated with lower risk of ischemic complications but higher risk of a bleeding complication. Outcomes research has revealed that the elderly have been treated less effectively ^12^. Presumably, practitioners consider the risk-benefit ratio of cardiac procedures to be less favorable in the elderly. While elderly patients did not share in the improved survival rates observed in younger patients in the early days of coronary interventions (1979–1994) ^2^, more recent data showed that mortality after hospital admission of elderly patients with acute myocardial infarction has substantially decreased over the past 15 years ^13^. This improvement is likely mediated by increasing use of recommended management strategies; thus, the application of guidelines derived from trials mostly including younger patients may benefit the elderly populations as well. However, adverse outcomes increase with age across the whole spectrum of ACS ^10, 14– 16^. Observational data suggest that outcomes of elderly patients improve when they receive guideline-directed cardiac procedures ^17^. Registry data from ACS events in England and Wales between 2003 and 2010 show substantial reduction in in-hospital mortality in all age groups, including the old and very old, males and females, and STEMI and non-STEMI. Within the study time frame, the use of percutaneous coronary intervention (PCI) and evidence-based pharmacologic therapies increased significantly for all age groups. Although elderly patients still received fewer coronary revascularizations than younger patients, it is noteworthy that almost half of all STEMI patients older than 85 years received a PCI, and the adherence to evidence-based pharmacotherapy at discharge was about 90% in this group ^18^. The Italian Elderly ACS study suggests that elderly patients with troponin elevation benefit from an early invasive approach ^19^. Data from the same trial, when pooled with registry data, suggested that coronary revascularization in elderly women was associated with lower 1-year mortality when compared with an early conservative approach, without an increase in severe bleeding ^20^. The results from the After Eighty study support the use of an invasive strategy in patients at least 80 years old ^21^. Even in nonagenarians and centenarians with ACS, there is evidence that adherence to guideline-recommended therapies is associated with decreased mortality ^22^. Despite an increased risk for major bleeding in patients older than 75 years of age, a routine early invasive strategy significantly improved outcomes in elderly patients with ACS ^23^. Registry data suggest that, over the last 15 years, the progressive switch from a conservative to a more invasive approach in elderly patients may have contributed to mortality reduction across the ACS spectrum, irrespective of age and gender ^24, 25^. Accordingly, the absolute benefit of early invasive therapies in the elderly appears to be greater than in younger patients because of their high mortality at baseline.

## Pharmacologic considerations

It has been reported that the risk of adverse drug reaction increases with the number of medications taken concurrently. With two concurrent medications, there is a 13% risk of an adverse drug interaction, and the risk increases to 38% for four medications and 82% for seven or more medications prescribed simultaneously ^26^. In light of these data, it is crucial to balance the risks of polypharmacy with the benefit of not withholding guideline-directed medications, proven to be of benefit in the elderly.

Age-related decline in organ function, muscle mass, and volume of distribution and changes in pharmacokinetics require the treating physician to meticulously adjust medication dosing ( Table 1). As bleeding risk increases with age, dose adjustments are particularly important when it comes to anticoagulant therapies. The creatinine clearance is preferably calculated by the Cockcroft-Gault equation and should form the basis for renally dosed medications rather than the serum creatinine level. Observational data revealed that patients with ACS often receive antithrombotic therapies at excess doses. Factors associated with excess dosing included older age as well as female sex, renal insufficiency, low body weight, diabetes mellitus, and CHF ^27^. Among patients who recently had an ACS, a high-dose statin regimen is known to provide greater protection against death or major cardiovascular events than a low- or moderate-dose statin regimen ^28^. A subgroup analysis from the PROVE-IT-TIMI 22 (Pravastatin or Atorvastatin Evaluation and Infection Therapy–Thrombolysis in Myocardial Infarction 22) trial including 634 elderly patients suggested that a high-dose statin regimen achieved a greater reduction in adverse events in the elderly than in the younger study subjects. Not only was the incidence of major statin side effects among the elderly similar to that in younger patients, it also did not differ with the intensity of the statin regimen ^29^. In similar fashion to the general ACS population, optimal medical therapy should be initiated before hospital discharge, as this strategy has been shown to improve long-term outpatient adherence. While randomized data are sparse in patients older than 75 years, an observational study has shown that early beta-blocker therapy was not used for 51% of patients at least 65 years old who were hospitalized with an acute myocardial infarction, although they did not have a clear contraindication to this therapy. Yet the same study showed that patients who received beta blockers had a lower in-hospital mortality rate than patients who did not receive beta blockers ^30^. Results from the CRUSADE (Can Rapid Risk Stratification of Unstable Angina Patients Suppress Adverse Outcomes With Early Implementation of the American College of Cardiology/American Heart Association Guidelines) National Quality Improvement Initiative have shown that early in-hospital use of aspirin and beta blockers was less likely in patients past 65 years of age and that heparin was significantly less used past 85 years of age. The acute use of clopidogrel and platelet glycoprotein IIb/IIIa inhibitors was most affected by age. Only 30% of patients older than 85 years received clopidogrel, and only 12.8% received platelet glycoprotein IIb/IIIa inhibitors. For patients surviving the index hospitalization, use of many discharge medications was similar in young and old patients except that clopidogrel and lipid-lowering therapy remained less commonly prescribed in elderly patients. While in-hospital mortality and complication rates increased with advancing age, those receiving more recommended therapies had lower mortality than those who did not ^31^.

**Table 1.** Acute coronary syndrome medications.

Antiplatelet
Aspirin
*P _2_Y _12_ antagonists*
Clopidogrel ^a^
Prasugrel ^a, b^ (relatively contraindicated in patients who are at least 75 years old or whose body weight is less than 60 kg)
Ticagrelor
Cangrelor ^b^
*Glycoprotein IIb/IIIa inhibitors*
Tirofiban ^b, c^
Eptifibatide ^b, c^
Abciximab ^b^
Anticoagulant
Heparin ^b^
Enoxaparin (low-molecular-weight heparin) ^a, b, c^
Dalteparin (low-molecular-weight heparin) ^b^
Fondaparinux ^c^ (in the US, use for acute coronary syndrome is off-label)
Argatroban ^b^
Bivalirudin ^b, c^ (vascular access site bleeding more often in patients older than 65 years)
Anti-ischemic
Nitroglycerin
Beta blocker ^c^ (in the elderly, consider lower initial doses and titrate to response)
Adjunct
Statins ^c^
Angiotensin-converting enzyme inhibitor ^c^
Angiotensin receptor blocker ^c^

^a^Need for age-based dose adjustment. ^b^Need for weight-based dose adjustment. ^c^Need for renal-based dose adjustment.

## Revascularization

Most myocardial infarctions in older adults present without ST elevations on the ECG and this has been attributed to the presence of more severe multivessel CAD that may have led to ischemic preconditioning or significant collateral growth ^32^. Less than 30% of ACS presentations in patients older than 75 years are caused by STEMI ^9^. While it is well established that coronary reperfusion is crucial within 12 hours of symptom onset, in the Global Registry of Acute Coronary Events (GRACE) registry, 30% of STEMI patients presenting within 12 hours of symptoms did not receive reperfusion therapy. Being 75 years of age or older was among the characteristics of those less likely to receive revascularization ^33^.

Current data support the use of reperfusion therapies, including fibrinolysis, up to the age of 85 years ^14^. The selection between fibrinolytics or PCI is determined largely by factors other than age, such as time from presentation, travel time to cardiac catheterization laboratory, comorbidity, and signs of cardiogenic shock ^34^. The safety and efficacy of reperfusion, specifically fibrinolytic therapy, in the very elderly (≥85 years of age) have yet to be determined as the risks of intracranial bleeding and cardiac rupture increase with age.

When revascularization options for the elderly are being considered, it is reasonable to choose coronary artery bypass graft (CABG) surgery over PCI in older patients with non-ST segment elevation ACS who are appropriate candidates, particularly those with diabetes mellitus or complex multivessel CAD, to improve outcomes ^11^. CABG may become the only option in certain clinical settings, such as a coronary anatomy not amenable to PCI, unsuccessful PCI, or the need for surgical repair of mechanical complications or concomitant valve disease.

When the decision is made for an invasive approach, the transradial access may be particularly appealing in the elderly as it reduces the risk of access site bleeding complications. In a subgroup analysis of the RIVAL (Radial Versus Femoral Access for Coronary Intervention) trial, elderly patients undergoing cardiac catheterization had lower rates of major bleeding or access site complications, yet the elderly also had higher rates of access site crossover with radial access compared with femoral access ^35^. Operative mortality rates and risk of complications for CABG increase with age. In addition, duration of hospitalization and post-surgery convalescence may be significantly prolonged in older patients after CABG and therefore should be considered in counselling the patient. It is up to the clinician to individualize the patient assessment by acknowledging the wide heterogeneity that exists between chronological and biological age and determining the patient’s preferences and goals for life. Another underutilized measure in the care of elderly patients with ACS is referral to cardiac rehabilitation. Given the overall reduced functional status in the older adult, the elderly are at an elevated risk of disability following ACS. There is evidence that the benefit of cardiac rehabilitation applies to both the elderly and younger coronary patients ^36^. Older adults may even benefit more, as cardiac rehabilitation in the elderly may extend beyond the cardiovascular system and include an improvement in physical fitness as well as enhancement in balance, stability, muscle strength, and tone.

## Conclusions

As the population continues to age, physicians will be confronted with an increasing number of elderly and very elderly patients presenting with ACS. While care needs to be individualized, age alone should never be the reason to withhold potentially lifesaving procedures and interventions. Elderly patients are at high risk for bleeding complications, but they are also at the highest risk for ischemic complications if less aggressive treatment strategies are pursued. So clinicians are tasked with meticulous risk stratification for ischemic risk and bleeding risk while taking into account assessment of frailty, quality of life, goals of care, and individual preferences. Early invasive protocols seem to be just as feasible in the elderly as in the general population. In order to mitigate bleeding risk and adverse medication side effects, it is imperative to correct dosages of pharmacotherapy to age- and gender-adjusted renal function and volume of distribution.

## Abbreviations

ACS, acute coronary syndrome; CABG, coronary artery bypass graft; CAD, coronary artery disease; CHF, congestive heart failure; ECG, electrocardiogram; LBBB, left bundle branch block; PCI, percutaneous coronary intervention; STEMI, ST segment elevation myocardial infarction.
